# A Paradigm of Uphill Running

**DOI:** 10.1371/journal.pone.0069006

**Published:** 2013-07-10

**Authors:** Johnny Padulo, Douglas Powell, Raffaele Milia, Luca Paolo Ardigò

**Affiliations:** 1 Tunisian Research Laboratory "Sports Performance Optimization" National Center of Medicine and Science in Sport, Tunis, Tunisia; 2 Faculty of Medicine and Surgery, University of Tor Vergata, Rome, Italy; 3 Department of Physical Therapy, Campbell University, Buies Creek, North Carolina, United States of America; 4 Faculty of Medicine and Surgery, University of Cagliari, Cagliari, Italy; 5 School of Exercise and Sport Science, Department of Neurological, Neuropsychological, Morphological and Movement Sciences, University of Verona, Verona, Italy; University of Sydney, Australia

## Abstract

The biomechanical management of bioenergetics of runners when running uphill was investigated. Several metabolic and mechanical variables have been studied simultaneously to spread light on the locomotory strategy operated by humans for effective locomotion. The studied variables were: heart rate, heart rate variability, oxygen intake and blood lactate, metabolic cost, kinematics, ground reaction force and muscular activity. 18 high-level competitive male runners ran at 70% VO_2max_ on different uphill slope conditions: 0%, 2% and 7%. Modifications were significant in almost all variables studied, and were more pronounced with increasing incline. Step frequency/length and ground reaction force are adjusted to cope with both the task of uphill progression and the available (limited) metabolic power. From 0% to 7% slope, step frequency and ground reaction force and metabolic cost increased concurrently by 4%, 12% and 53%, respectively (with a 4% step length decrease as well). It is hypothesised that this biomechanical management is allowed by an environment-body communication performed by means of specific muscular activity.

## Introduction

Running as a form of human locomotion has often interested exercise physiologists and biomechanists, who aimed to increase their knowledge and understanding of its featuring variables [1], [2]. In recent years, there has been an expansion of research with regard to both biomechanics [3] and metabolic cost [4] of running. There are many factors that affect running performance, including environmental and geographical factors. It has been established that environmental factors such as dehydration [5] and hyperthermia [6] can alter physiological performance. Geographical factors relate to geomorphology and the variations in surfaces and terrain, including the slope of the running surface. It is evident that there are differences in mechanical variables between level and uphill running, in which alterations are required to adapt to the environmental circumstances. Research has suggested uphill running is associated with increased metabolic cost [4], [7]. Research has identified that as metabolic cost increases, decreases in step length with concomitant increases in step frequency are adopted to maintain constant speed during uphill running [3].

Although training with sloped surfaces is used by many coaches as specific strength training, slight sloping surfaces are often evident within endurance races [3]. It is therefore pertinent to investigate the mechanisms of adaptation required during uphill running. It has been suggested that when running on level surfaces runners use technique optimized for minimal metabolic cost; however, when the inclination of the surface is altered runners will modify mechanical variables to achieve optimal metabolic efficiency. Currently, the strategies underlying increased step frequency and decreased step length in uphill, constant velocity running are not well established. Previous research has revealed that uphill running is associated with greater energy expenditure [8], increases in step frequency and decreases in ground reaction forces (GRF) [9].

Previous studies investigating changes in GRF [9] and metabolic cost [10] of running on sloped surfaces have investigated these variables independently. There is dearth of literature that has investigated the increases in oxygen consumption with respect to the biomechanical variables underlying these increased metabolic demands. There is a clear interplay between step frequency, step length, ground reaction force and determine metabolic cost, especially during uphill running. The relationships among these variables may be mediated by a differential commitment of the nervous system as well [11]. An excessive step frequency with shortened step length may increase metabolic cost through an increased mechanical kinematic internal work [12]. An excessively low step frequency combined with elongated step length may result in greater ground reaction forces and a consequent increased metabolic cost. It can be speculated that the adopted strategy seeks to optimize step frequency, step length and ground reaction force in order to allow an effective uphill running. However, the interaction of biomechanical and metabolic parameters by which this optimal strategy is selected remains sparsely investigated.

Therefore, the aim of this study is to investigate the relationship between metabolic and biomechanical variables simultaneously, in order to describe the strategies employed during uphill running to optimize metabolic expenditure and consequently performance.

## Methods

### Participants

Eighteen male marathon runners participated in this study (age 33.0±8.5 [mean ± SD] years, mass 62.6±5.2 kg, height 1.71±0.04 m, BMI 21.4±1.0 kg/m^2^). Written informed consent was obtained prior to data collection, and the study was approved by the Ethics Committee of the University of Tor Vergata, Rome, ITALY (protocol no. 112-A2-2011). All procedures were performed in accordance with the Declaration of Helsinki on the use of human subjects. The inclusion criteria were: high level running (all were ranked at the amateur national level with best marathon race times varying from 2∶40 to 2∶50 h:min). The subjects’ training background consisted of 11.0±1.1 years, running 151.0±6.3 km/week in the latest year and a VO_2max_ of 76.3±2.6 ml·min^−1^·kg^−1^. The VO_2max_ was measured by means of a standard Åstrand treadmill incremental protocol one week before data collection. The subjects were healthy, with no muscular, neurological and tendon injuries and were clear of any drug consumption. All subjects were homogeneous with regard to their training status and none of the subjects underwent any strenuous endurance activity and/or resistance training outside their normal endurance training protocol.

### Procedure

The assessment was divided into two days (separated by three days) and was conducted as follows: (1) uphill running conditions on a treadmill (kinematics and metabolics), (2) over-ground uphill running conditions (kinematics and kinetics). Initial tests were conducted in the Human Performance Laboratory. Tests included analysis of heart rate (HR), heart rate variability (HRV), oxygen intake and blood lactate, kinematic variables and electromyography (EMG). Data were collected during a single session, between 3∶00 p.m. and 7∶00 p.m. under an average temperature of 23°C (min 20°C, max 26°C). All subjects wore running shoes (Category A3) and performed a standardized 15-minute warm-up, consisting of a run at 9 km·h^−1^ to familiarise themselves with the treadmill [13] (Run Race Technogym® Run 500, Gambettola Italy).

The treadmill was set at 0% (0°), 2% (1°), 7% (4°) incline for five minutes per each condition at a constant velocity. The treadmill incline/velocity setting was calibrated before each test according to the instructions of the manufacturer and regularly checked after the test. Percent grade was expressed as being equal to the tangent [theta] × 100. The experiment started using a randomised protocol (Latin square design for one speed and three slopes) at 4.17 m·s^−1^ (70% of the VO_2max_ velocity) at 0% followed by both slope conditions (2% and 7%). Following each condition, a 5 min passive recovery session was utilised, which is in accordance with the protocol proposed by Cavanagh et al [14]. The protocol included the following: 5 min running at 4.17 m·s^−1^ at 0% incline, followed by 5 min passive recovery. Testing resumed with 5 min running at 4.17 m·s^−1^ at 2% incline, followed by 5 min passive recovery. Then 5 min at 4.17 m·s^−1^ at 7% incline were completed, followed by 5 min passive recovery. During testing, the procedure was never interrupted and the subjects were not injured.

### Heart Rate

Heart rate was recorded throughout the experiment, computed beat to beat, using a Polar S810 heart-rate monitor (HRM; Polar Electro OY, Kempele, Finland). After data acquisition, heart rate variability (HRV) was calculated using Kubios Hrv software (Department of Physics, University of Kuopio, Finland). Frequency domain measures of HRV were derived by fast Fourier transformation: they were both low-frequency (LF; 0.04–0.15 Hz) and high-frequency (HF; 0.15–0.40 Hz) spectral power.

### Oxygen Intake, Blood Lactate and Metabolic Cost

The subjects’ oxygen consumption (VO_2_) was measured using a breath-by-breath metabolic measurement system (Med-Graphics Breeze, St Paul, MN, USA) during the treadmill protocol. The system was calibrated immediately prior to each exercise test on the treadmill. Peak blood lactate (BLa) concentration (mmol·L^−1^) was determined at the end of each run by means of serial samples. Micro samples of arterialised blood from the ear lobe were taken and immediately analysed with a lactate analyser: Arkray Lactate Pro LT-1710 analyser (whole blood) (Arkray Inc. Kyoto, Japan) [15]. Metabolic cost (C_r_) was calculated following Di Prampero’s approach [4]. Resting VO_2_ and BLa were assumed as 3.5 ml·min^−1^·kg^−1^ and 1 mmol·L^−1^, respectively. Net VO_2_ was considered as representative of the effective aerobic metabolic power. BLa accumulation was considered as representative of the effective anaerobic lactic power and converted into its corresponding VO_2_ by multiplying it by the conversion factor of 3 ml·min^−1^·kg^−1^ (mmol·L^−1^) ^−1^. The anaerobic a-lactic power was considered negligible. C_r_ is defined as the overall metabolic energy required above resting to transport the subject’s body over one unit of distance [4]. Throughout this paper it is expressed in joules per kilogram per meter on the assumption that 1 ml O_2_ consumed in the human body yields 20.9 J (which is strictly true only if the respiratory quotient = 0.96) [4].

### Kinematic Analysis

Two-dimensional (2D) running kinematic data were captured using a high speed camera (Casio Exilim FH20) with a sampling rate of 210 Hz. In accordance with other studies [3], [11], [16], [17], considering that the treadmill platform was 50 cm high, the camera was positioned on a 1.5 m high tripod, 6 m from the participant and was located perpendicular to the plane of motion and the participant’s sagittal plane [18] as standard calibration. The film sequences were analysed off-line using Dartfish 5.5 Pro motion analysis software (Dartfish, Fribourg, CH). The following kinematic variables were studied: (i) contact time (ms), (ii) flight time (ms), (iii) step length (m), and (iv) step frequency (Hz). 400 steps were sampled [19]. Since the velocity of the treadmill was known, both step length (SL) and step frequency (SF) could be calculated. The contact time (CT) and flight time (FT) were calculated by counting the frames in contact and flight on the 2D data, then dividing by the sampling rate, 210 (1 frame = 210 Hz ≈ 0.0048 sec). The resolution was 480 × 360 for a spatial precision of about ±4.5 millimetres.

The CT and FT were calculated for both the left and right foot. The CT was defined and calculated as the time between initial contact with the ground and the last frame of contact before toe-off. The FT was defined and calculated as the time between toe-off and subsequent initial contact of the contra-lateral foot. Initial contact and toe-off were visually detected. In accordance with previous studies [3], [11], [16], [17], [20], SF was calculated as SF = [1000/(CT+FT)], SL was calculated with the following equation SL = [speed m·s^−1^/SF]. The test–retest reliability of this testing procedure was demonstrated through an Intra-class Correlation Coefficient (ICC) and standard error of measurements (SEM) for the following variables: SL (ICC: 0.95–0.98, SEM: 0.05–0.08 m), SF (ICC: 0.95–0.98, SEM: 0.11–0.13 Hz), CT (ICC: 0.96–0.98, SEM: 12–15 ms), and FT (ICC: 0.95–0.98, SEM: 11–15 ms).

### Electromyography Analysis

EMG activity of the tibialis anterior (TA), vastus lateralis (VL), rectus femoris (RF), gastrocnemius medialis (GM), biceps femoris (BF) and gluteus major (MG) of the right leg were collected [21]. Active bipolar electrodes (inter-electrode distance 1.2 cm) were aligned along the fibres of the muscle under investigation according to the recommendations by SENIAM [22]. Prior to electrode placement, each site was shaved, cleansed with alcohol and gently abraded, and a small amount of conductive gel was applied to each electrode to minimize impedance. In order to reduce cable movement artefact, cables were secured using elastic bands (Vetrap™) [23]. An amplifier (gain × 600, input impedance 2 GΩ, common-mode rejection ratio 100 dB, band-pass filter 6–1500 Hz; Biochip Grenoble, France) was used [24]. The Muscle Lab Encoder converted the amplified EMG raw signal to root mean square (RMS) signal total error ±0.5%. EMGrms was expressed as a function of time (mV) and calculate (peak-to-peak) as mean percentages within the three different conditions (0, 2, 7% inclines). Furthermore, the goniometric data (MuscleLabTM 4020e, Bosco System, Ergotest Technology, Langensund, Norway) were synchronised with the EMG signals into a synchronised videotape with MuscleLab System. A personal computer (Sony Vaio TT21WN) was used to collect and store the data. The summed EMGrms of the six muscles was used for statistical analysis.

### Ground Reaction Force

**Figure 1:**
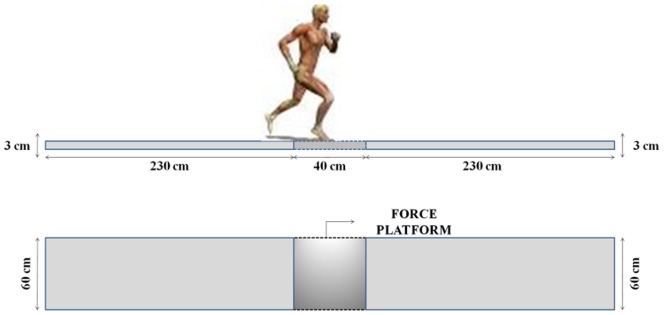
Position of the force platforms with relation to the runway.

On the second day of testing, all participants performed various tests on sections of 50 m asphalt road at the following inclines 0%, 2%, and 7%. Incline was calculated as being equal to the tangent [theta] × 100, every five meters per metric with a constant slope and monitored every 10 m with respect to a beep with default speeds up to the target velocity of 4.17 m·s^−1^. After a 20-minute warm-up, the participants performed nine test runs, using a standing start. The time between the runs (1 minute) was sufficient for the participants to recover fully. All participants performed several tests with increasing speed and slope. Three trials for each participant were used in order to establish the magnitude of variability associated with repeated trials. Ground reaction force (GRF) data were collected at 500 Hz with one force platform (Model 9281A, Kistler AG, size 0.4×0.6 m), which was mounted in the middle part of the runway (Figure 1). Photocells were set at 5 m before (first pair) and 5 m after (second pair) the force platform in order to measure the elapsed time to run the 10 m section. 2D kinematic data analysis (CT, FT, SF, SL) of 20 cycles were conducted at each slope condition, in which the set-up was the same as during the treadmill run including: high speed video data were collected at 210 Hz (Casio Exilim, FH20), set on a 1.5 m high tripod, 6 m from the participant and perpendicular to the subjects’ sagittal plane. Video sequences were analyzed off-line using Dartfish 5.5 Pro (Dartfish, Fribourg, CH).

### Statistical Analyses

Descriptive statistics are presented as mean (± SD). Statistical analysis was performed by using SPSS software (version 15, SPSS Inc., Chicago, IL, USA). The sample size was determined with *post hoc* statistical power analysis with G-Power 3.1.3. Using the statistical power of ANOVA by SPSS we calculated the total sample size with G-Power 3.1.3. For testing the repeatability of the kinematic measure, we performed an intra-class correlation coefficient (ICC) [25]. Linear regression analysis, using Pearson’s correlation coefficients (r), was used to indicate strength of the relationship between incline and velocity. After the assumption of normality was verified, using the Kolmogorov-Smirnov test, one-way analysis of variance (ANOVA) was used to determine any significant differences between all variables. *Post hoc* tests were conducted when significant main effects were found using Fisher's least significant difference (LSD). Significance level was set at *P*≤0.05.

## Results

Heart rate at 0% was 148.0±12.2 beats·min^−1^, which increased by 5.06% at 2% up to 155.0±12.0 beats·min^−1^ (r = 0.964, 95% confidence interval [CI]: 0.79–0.99, *P*<0.001). Relatively to 0% gradient, heart rate increased by 15.12% up to 170.0±11.8 beats·min^−1^ at 7% (r = 0.938, CI: 0.65–0.99, *P*<0.001). A 9.58% increase (r = 0.924, 95% CI: 0.69–0.98, *P*<0.001) occurred from 2% to 7% incline (Table 1; *F* = 1226.194 *P*<0.001).

**Table 1:** Effects of uphill treadmill running on cardiovascular variables.

Condition	0% Run	0% Recovery	2% Run	2% Recovery	7% Run	7% Recovery
**HR (beats·min^−1^)**	148±12.21	96±3.77	155±12.02	95±1.54	170±11.37	114±2.19
**R-R (s)**	0.388±0.02	0.628±0.02	0.327±0.05	0.588±0.07	0.280±0.05	0.557±0.05
**LF (Hz)**	0.0911±0.04	0.0768±0.03	0.0820±0.05	0.0827±0.03	0.0833±0.04	0.0723±0.04
**HF (Hz)**	0.2721±0.09	0.2350±0.09	0.3158±0.06	0.2409±0.11	0.2728±0.08	0.1829±0.03

Values are presented as mean and standard deviation. Abbreviations: heart rate (HR), R-R beat to beat interval (R-R), low-frequency heart rate variability (LF), high-frequency heart rate variability (HF).

Inter-beat interval (R-R) during running at 0% was 0.39±0.02 s and during subsequent recovery was 0.63±0.02 s, resulting thus with a difference of +61.73% (Table 1; *F* = 1389.319 *P*<0.001). At 2% gradient, R-R was 0.33±0.05 s showing thus a decrease of −14.44±11.21% with respect to running at 0% (*P*<0.001). During recovery at 2% gradient, R-R was 79.66% greater than running at 2%, eliciting a value of 0.59±0.07 s. At 2% incline, with respect to 0% incline, HRV low-frequency (LF) decreased by 10%; HRV high-frequency (HF) however increased by 16.03%. R-R during running at 7% was 0.28±0.05 s, showing thus a decrease of 27.84% and 14.37% with respect to running at 0% and 2%, respectively. During recovery after 7% incline run, phase R-R increased by 98.74% compared to 7% running, eliciting a value of 0.56±0.05 s. At 7% incline running, LF decreased by 8.57%, while HF increased by 0.24% (Table 1).

**Figure 2:**
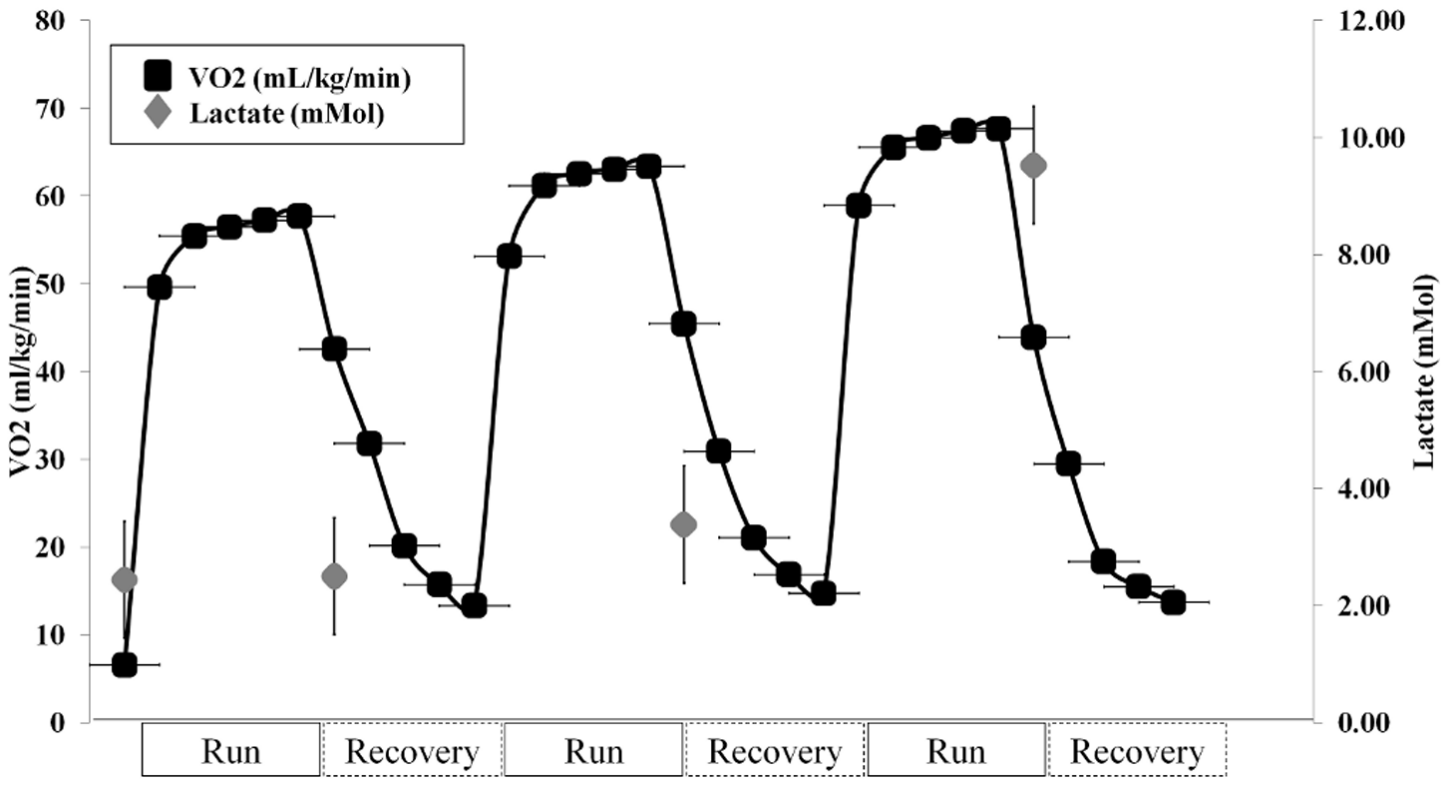
VO2 throughout the duration of the test: 5 minutes running and 5 minutes of recovery at 0%, 2% and 7% slopes, respectively. For each condition the first lactate value represents baseline, followed by each subsequent minute within the condition. Data are expressed at mean and error bars. VO2 error bars are horizontal purely for graphical purpose.

Average VO_2_ during 0% treadmill running was 54.6±6.6 ml·kg^−1^·min^−1^, while VO_2_ increased by 10% up to 60.0±7.5 ml·kg^−1^·min^−1^ when running on a 2% incline. The increase was significant (*P*<0.001). Relative to 0%, running at 7% incline elicited a 19% significant increase (*P*<0.001) with a VO_2_ of 64.8±6.2 ml·kg^−1^·min^−1^ (Figure 2; *F* = 17.768 *P*<0.001). Blood lactate showed a similar trend. Average BLa during running at 0% incline was 2.50±0.89 mmol·L^−1^ (Figure 2; *F* = 38.066 *P*<0.001), while it increased by 35.5% (*P* = 0.07) up to 3.39±1.48 mmol·L^−1^ at 2% incline. Relatively to 0%, during running at 7% incline, average blood lactate significantly increased up to 9.53±2.26 mmol·L^−1^ (*P*<0.001). The overall C_r_ (resulting from the sum of the effectively measured VO_2_ and the accumulated BLa corresponding VO_2_; see Oxygen intake, blood lactate and metabolic cost) amounted to 4.91±0.41 J·kg^−1^·m^−1^ at 0%, 5.61±0.38 J·kg^−1^·m^−1^ at 2% (+14% with respect to 0%, significant, *P*<0.001), and 7.51±0.56 J·kg^−1^·m^−1^ at 7% (+53% with respect to 0%, significant, *P*<0.0001; +34% with respect to 2%, significant, *P*<0.0001; ANOVA *F* = 181.310 *P*<0.001).

Kinematic variables (CT, FT, SF, SL) during both treadmill and over-ground running are presented in Table 2 (CT *F* = 1.933 *P* = 0.161; FT *F* = 5.313 *P* = 0.01; SF *F* = 12.141 *P*<0.001; SL *F* = 12.009 *P*<0.001) and 3 (CT *F* = 0.839 *P* = 0.441; FT *F* = 20.712 *P*<0.001; SF *F* = 10.883 *P*<0.001; SL *F* = 11.298 *P*<0.001), respectively, while GRFs are presented in Table 3 (Force [N] *F* = 1.025 *P* = 0.370; F/Bw [N/kg] *F* = 1.205 *P* = 0.313; Impulse [N/kg ms] *F* = 0.114 *P* = 0.893).

**Table 2:** Effects of uphill treadmill gradient on running kinematic variables.

Condition	0%	2%	7%	0–2 (Δ %) *P*	2–7 (Δ %) *P*	0–7 (Δ %) *P*
**Step length (m)**	1.41±0.04	1.39±0.04	1.35±0.03	−1.81% ***P*** ** = 0.013**	−2.53% ***P*** ** = 0.027**	−4.30% ***P*** ** = 0.0001**
**Step frequency (Hz)**	2.95±0.09	3.01±0.09	3.09±0.07	1.85% ***P*** ** = 0.014**	2.56% ***P*** ** = 0.024**	4.46% ***P*** ** = 0.0001**
**Flight time (ms)**	156±22.59	153±22.16	135±19.83	−2.29% *P* = 0.180	−11.70% *P* = 0.065	−13.72% ***P*** ** = 0.003**
**Contact time (ms)**	183±14.48	180±14.06	189±15.11	−1.41% *P* = 0.627	5.24% *P* = 0.165	3.76% *P* = 0.069

Values represent mean and standard deviation for all subjects (*n* = 18). Percentage difference between slope conditions are presented: significant differences between conditions are highlighted in **bold**.

**Table 3:** Changes in kinematic and GRF variables when running uphill (on ground).

Condition	0%	2%	7%	0–2 (Δ %) *P*	2–7 (Δ %) *P*	0–7 (Δ %) *P*
**Step length (m)**	1.46±0.10	1.42±0.08	1.39±0.07	−2.80% *P* = 0.452	−2.10% *P* = 0.414	−4.84% *P* = 0.131
**Step frequency (Hz)**	2.87±0.21	2.95±0.18	2.98±0.16	2.87% *P* = 0.372	1.01% *P* = 0.705	3.90% *P* = 0.213
**Flight time (ms)**	178±17.30	166±13.58	154±12.28	**−6.29% ** ***P*** ** = 0.008**	**−7.44% ** ***P*** ** = 0.001**	**−13.26% ** ***P*** ** = 0.0001**
**Contact time (ms)**	174±7.70	171±5.20	179±5.20	−1.40% *P* = 0.763	4.18% *P* = 0.330	2.72% *P* = 0.493
**Force (N)**	1266±3.68	1332±3.51	1419±4.13	5.19% *P* = 0.354	6.53% *P* = 0.160	**12.06% ** ***P*** ** = 0.030**
**F/Bw (N/kg)**	19.93±2.54	21.04±2.0	22.38±2.46	5.57% *P* = 0.613	6.39% *P* = 0.403	12.32% *P* = 0.191
**Impulse (F/kg ms)**	3461±515	3594±329	3970±447	3.84% *P* = 0.579	**10.44% ** ***P*** ** = 0.006**	**14.69% ** ***P*** ** = 0.002**

Values represent mean and standard deviation for all subjects (*n* = 18). Percentage difference between slope conditions are presented: significant differences between conditions are highlighted in **bold**.

**Figure 3:**
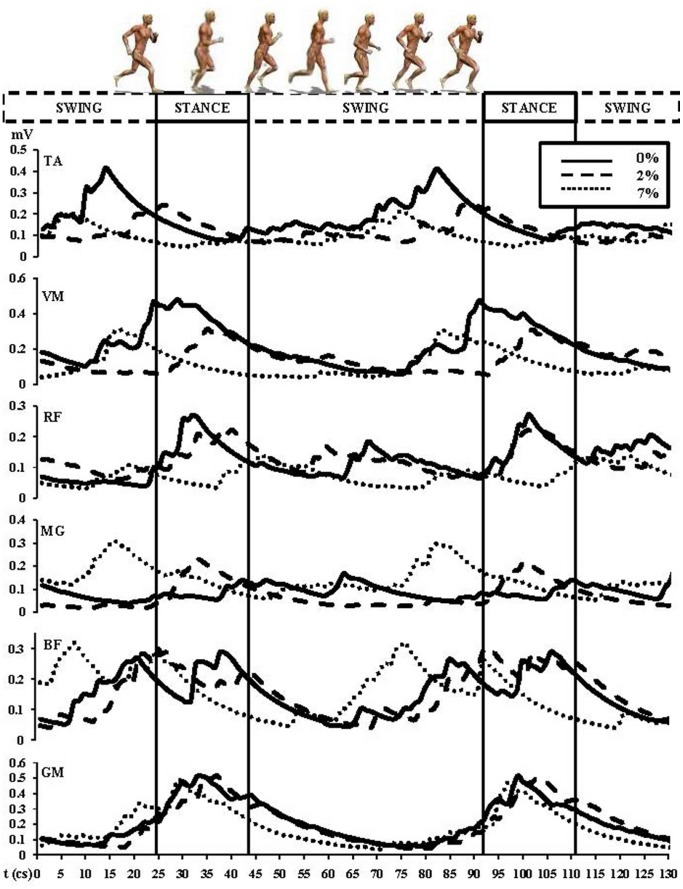
Rectified and smoothed EMG curves indicate electrical activity of the lower limb muscle groups at different slopes 0%, 2% and 7% and during different phases of gait (stance and swing). Abbreviations: tibialis anterior (TA), vastus medialis (VM), rectus femoris (RF), gluteus major (MG), biceps femoris (BF) and gastrocnemius medialis (GM).

The linear envelope EMGrms patterns were similar in each locomotion condition, with all muscles active before and during stance. The most striking significant statistical evidences following EMGrms ANOVA with LSD (Figure 3; TA *F* = 62.209 *P*<0.001; RF *F* = 54.243 *P*<0.001; VM *F* = 44.151 *P*<0.001; GM *F* = 2.701 *P* = 0.068; MG *F* = 68.482 *P*<0.001; BF *F* = 0.031 *P* = 0.970) were large changes in magnitude during the slope runs, during which activity significantly reduced with increasing incline for the following muscles: TA (0–2% = −43.42%, *P*<0.001; 0–7% = −48.81%, *P*<0.001; negative correlation, r = −0.425, 95% interval confidence: −0.87–0.40, *P*<0.001), RF (0–2% = −16.25%, *P* = 0.891; 0–7% = −47.75%, *P*<0.001; negative correlation, r = −0.449, 95% interval confidence: −0.88–0.37, *P*<0.001), VM (0–2% = −35.22%, *P*<0.001; 0–7% = −37.14%, *P*<0.001; negative correlation, r = −0.376, 95% interval confidence: −0.85–0.45, *P*<0.001). The GM remained relatively consistent between 0% and 2%, however it elicited a slight reduction at 7% (0–2% = 0.09%, *P* = 0.430; 0–7% = −4.16%, *P* = 0.02; negative correlation, r = −0.116, 95% interval confidence: −0.76–0.64, *P* = 0.02). Conversely, EMG activity was elevated for the MG (0–2% = 41.42%, *P* = 0.02; 0–7% = 83.27%, *P*<0.001; positive correlation, r = 0.451, 95% interval confidence: −0.37–0.84, *P*<0.001). Similarly, the BF increased gradually as a function of the increasing treadmill gradient (0–2% = 0.79%, *P* = 0.891; 0–7% = 6.16%, *P* = 0.901; Figure 2).

## Discussion

Sloped running is associated with mechanical and metabolic adaptation. Runners adapt their neuromuscular strategy to optimize metabolic energy expenditure. The purpose of the present study was to investigate these altered strategies when running on different increasing slopes. In support of previous studies, the present study showed increases in metabolic variables in response to to increasing slope [10], including: heart rate, oxygen consumption blood lactate, and metabolic cost. These data confirm a trend dependent on the slope. Also associated with the increases in metabolic variables, increases in SF were observed including a 2.0% increase was evident between 0% and 2% and a 4.8% increase between 0% and 7%, with similar relative decreases in SL.

The uphill running strategies were associated with increased SF and CT, and concomitant decreases in SL and FT during both over-ground and treadmill running. Increased SF generates a greater metabolic demand, explaining the observed increases in oxygen consumption. SF is directly related to step time. Therefore, changes in relative step time contribution, (i.e., decreases in FT and increases in CT) occurred in response to increased SF. Similarly, the FT is closely linked to changes in the CT. As demonstrated by previous research, the reduction of CT enhances the FT and vice-versa [14], . The CT is the only time featured by contact with the surface. The foot contact could act also as means to provide (external) environment-body communication about foot placement and running surface inclination. Evidence supporting the importance of CT in determining mechanical and metabolic performance and efficiency can be found in the timing and magnitudes of lower extremity muscle activation. Firstly, the contact phase is clearly featured by an increased activity of the lower extremity extensors including the VM, RF, BF and GM (Figure 3 stance phase). This could be an indication not only of their mechanical function, but also of their role in sensorimotor integration as well. A mechanical outcome of foot contact, GRF, increased by 5.19% in response to a 2% slope, while a 7% slope elicited a 12.06% increase in GRF. This is a dynamic compulsory phenomenon due to the requisite need to develop supplementary vertical force greater than the resistance of body weight to create the upward acceleration imposed by the increasing elevation.

Also supporting the role of muscle length and activation in control of mechanical and metabolic strategies in uphill running is the enhanced activation of the TA and MG during the swing phase of running. During the swing phase, the contribution of the foot to the central pattern generator [28] is minimal and consists solely of kinematic information. However, slope-dependent changes in the orientation and position of the foot relative to the ground may influence lower extremity muscle activation patterns.

The MG and the BF increased their cycle activity with increased slope: MG (0–2% slope = 41.42%, 0–7% = 83.27%) and BF (0–2% slope = 0.8%, 0–7% = 6.16%). Their activity increases were clearly more pronounced at 7% (Figure 3). Therefore, a hypothetical model pertaining to the muscles’ involvement in the environment-body communication during gradient running could be the following one. VM, RF, BF and GM provide information during CT, with involvement by BF increasing over slope and with involvement by VM and RF decreasing over slope. Conversely, TA and MG provide information during FT, with involvement by MG increasing over slope and with involvement by TA decreasing over slope. An overall efferent feedback through neural mechanisms is represented by the HRV change (i.e., an increase over slope) ruled by neural centres and with reasons still unknown. Such an HRV response is present in cycling as well [29]. Further studies need to confirm our hypothetical model.

**Figure 4:**
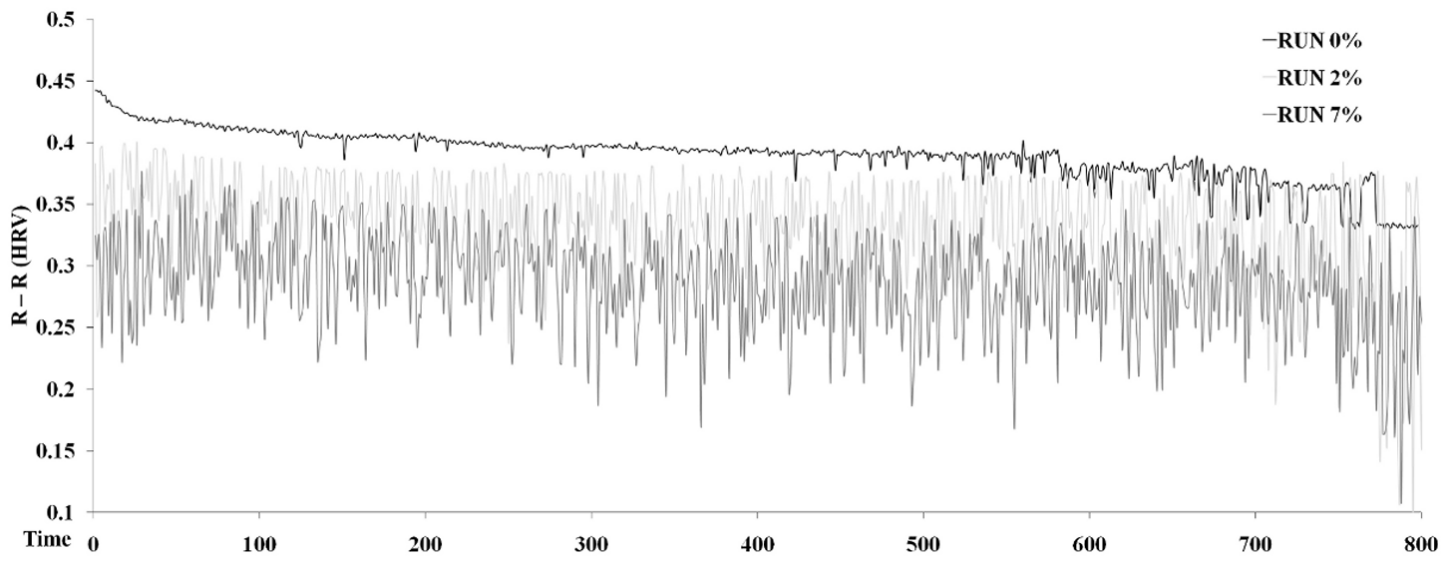
R-R intervals whilst running at 0%, 2% and 7% slopes. The graph represents the R-R variation throughout the duration of each condition.

HRV data further support the role of sensorimotor feedback in determination of optimal metabolic and mechanical strategies. HRV is suggested to be indicative of underlying neural control mechanisms [30]. Emerging from dynamical systems theory, reduced HRV is associated with reduced cardiovascular adaptability and pathology. The present study demonstrates that HRV was significantly affected by the increased mechanical and metabolic demands of uphill running (Figure 4). Increases in slope produced decreases in cycle activation of the TA (0–2% slope = 43.42%, 0–7% = 48.81%), the VM (0–2% slope = 35.22%, 0–7% = 37.14%) and the RF (0–2% slope = 16.25%; 0–7% = 47.75%).

The current study shows that evident changes occur about many different investigated variables with changes in slope. A large number of variables have been targeted to try to shed light on the relationships between the mechanical and metabolic during uphill running. After reviewing relative changes, it has been confirmed that running at increasing slope elicits greater heart rate, metabolic and mechanical cost (Table 1, Figure 2).

The EMG and GRF data, however, showed less evident changes when running uphill, with relation to level running (Table 3, Figure 3). This leads to the observation that with constant speed, each subject increased SF with a consequent increasing ground contact burden to be increasingly managed by the neuromuscular complex. The increase of the metabolic cost in uphill running is also related with the increases in internal mechanical work (W_INT_), which in turn is related to the SF [31]. W_INT_ is the mechanical work related to the movement of the body segments with respect to the body centre of mass position. Therefore, the measure of SF is very useful both to monitor itself and to estimate W_INT_, which is a partial determinant of the increase of the metabolic cost. When conducting uphill running the CPG might modulate SF/SL and related GRF by using muscles as both efferent and afferent (about kinematics and dynamics) components. This view is supported by the fact that the GRF increase on the slope is kept minimal, like the SL, to allow for effective uphill progression without placing an excessive burden on the metabolic system. If SL/SF would not change properly during uphill running, the feasible thrust GRF would not be sufficient to cope with the progressive increases in required parameters kinetic [3].

Conversely, if more metabolic power would be available, a different SL/SF management and consequently developed GRF would allow for improved performance. In this regard, cycling provides an interesting corollary. While cycling man is bound to the seat. If handlebar and pedals would not change his kinematics, when cycling at constant speed with increasing slope, the metabolic requirement would easily increase by 66% [32], likely placing an insurmountable burden on the subject’s metabolic system. However, while cycling uphill, man adjusts his kinematics (i.e., pedalling frequency) to develop the required mechanical power and minimise a required metabolic power [33] increase down to 5.2% [34]. Furthermore, Padulo et al [17] showed that is possible to run uphill without increasing metabolic cost by means of adjusting SF.

In conclusion a concomitant biomechanical and bioenergetical investigation of uphill running provides indications about the strategy operated by trained athletes to manage the interplay between step frequency/length and ground reaction force to control the metabolic cost. The results prompt toward a hypothetical model about the environment-body communication taking place during both CT and FT. The main model’s actors were big lower limb muscles and their sensory outputs. The whole model would likely be under an overall supervision by higher neural centres. At present the suggested model is by far preliminary. The integrated kinematic, dynamic, electromyographic and metabolic investigation performed within this study should be applied also with other modes of legged endurance locomotion (e.g., mountain trekking) to effectively develop a working general model. The specific supervision role of higher neural centres should be investigated by using a proper neurophysiological approach. The functional meaning of the HRV response should be specifically investigated as well.

The study data are available upon email request to luca.ardigo@univr.it.
